# Recurrent pineal tumor in a young adult male: Challenges in diagnosis and multimodal treatment management

**DOI:** 10.1016/j.radcr.2025.03.011

**Published:** 2025-03-27

**Authors:** Lydia Kiwanuka, Induni Nayodhara Weerarathna, Neha Rahul, Manishimwe Jules, Anurag Luharia, Zahir Quazi

**Affiliations:** a Department of Medical Radiology and Imaging technology, Datta Meghe Institute of Higher Education and Research (Deemed to be University), Wardha, Maharashtra, 442001, India; b Department of Biomedical Sciences, Datta Meghe Institute of Higher Education and Research (Deemed to be University), Wardha, Maharashtra, 442001, India; c Department of Radio Oncology, Datta Meghe Institute of Higher Education and Research (Deemed to be University), Wardha, Maharashtra, 442001, India; d Department of Radiotherapy, Datta Meghe Institute of Higher Education and Research (Deemed to be University), Wardha, Maharashtra, 442001, India; e Department research and development, Datta Meghe Institute of Higher Education and Research (Deemed to be University), Wardha, Maharashtra, 442001, India

**Keywords:** Pineoblastomas, Pineal gland tumors, Craniotomy, Recurrent pineoblastoma, Cranial irradiation, Concurrent chemotherapy

## Abstract

Pineoblastomas are rare, severe primary brain tumors originating in the pineal gland. They might be challenging to detect and cure. This article recounts the example of a 23-year-old man who suffered from regular headaches and visual issues. During the first MRI, a solid cystic tumor in the pineal area was identified, creating obstructive hydrocephalus by squeezing surrounding brain regions. A low-grade glial tumor was discovered during the operation, which included partial tumor excision and an endoscopic third ventriculostomy. However, follow up imaging revealed a rapid recurrence requiring external ventricular drainage and a second craniotomy. Following the second surgery, histopathology confirmed pineoblastoma, demonstrating the aggressive character of the tumor. Postoperative imaging showed persistent illness despite these measures, requiring the implementation of a comprehensive treatment strategy. The multidisciplinary team suggested craniospinal irradiation (35 Gy in 21 fractions) followed by lesion boost (19.8 Gy in 11 fractions), using VMAT (Volumetric modulated Arc therapy) technique, along with concurrent chemotherapy followed by adjuvant chemotherapy. This case illustrates the difficulties in identifying and managing recurrent pineal tumors, including the need for appropriate adjuvant treatment, surgical constraints, and recurrence concerns. It provides crucial information regarding the challenges of treating aggressive brain tumors and emphasizes the need for interdisciplinary care to achieve the best results. This is a rare case of Central Nervous system recurrent tumor, which was earlier thought to be a low grade pineal tumor but later turns out to be high grade, Pineoblastoma in a young adult male. The case emphasizes the challenges in correct diagnosis of CNS tumors, importance of Immunohistochemistry and prompt management for complete cure of the disease.

## Introduction

A rare and extremely dangerous brain tumor, pineoblastoma develops from the pineal gland, a tiny endocrine organ in the brain that produces the hormone melatonin, essential to the body's regular sleep-wake cycle [1]. The World Health Organization (WHO) has classified it as a Grade IV tumor, indicating that it is high-grade and aggressive and falls under the category of primitive neuroectodermal tumors (PNETs). Pineoblastoma (PB) accounts for fewer than 0.1% of intracranial neoplasms and is more prevalent in youngsters than adults, with adult cases comprising less than 10% [2]. Pineal tumors are generally uncommon, accounting for only 0.2% of all brain tumors and having an age-adjusted incidence rate (AAIR) of 0.05 per 100,000. Black patients and children had the highest frequency of PB, while males, small children, elderly persons, and those who did not have surgery had the lowest survival rates [3].

Historically, pineal parenchymal tumors were categorized as either pineoblastoma, pineocytoma, or pineocytomapineoblastoma. Pineoblastomas are thought to be the most aggressive among the pineal parenchymal tumors. They have a poor prognosis and a high death rate during the first 5 years of diagnosis [4]. Regarding histology, PBs are infiltrative, weakly differentiated, and have a distinctively primitive neuroectodermal look. Although it seldom occurs outside the central nervous system (CNS), leptomeningeal dispersion is frequent [5]. Clinically, when PB interferes with cerebrospinal fluid (CSF) flow through the cerebral aqueduct, it can lead to hydrocephalus, a backup of CSF that raises intracranial pressure. Headaches, drowsiness, vomiting, and eye-light alterations are common patient symptoms [6].

Additionally, depending on the size of the tumor and its local invasion, other symptoms could include visual abnormalities, Parinaud's syndrome (a combination of eyelid retraction, convergence-retraction nystagmus, and upward gaze palsy), and unspecified neurological deficiencies [7]. Neuroimaging is essential in diagnosing, planning, and following up patients with pineal masses. Pineoblastomas typically manifest as irregular, poorly defined, lobulated tumors that are huge (more than 3 cm) and frequently invade the nearby brain. Their high cellularity causes them to appear hyperdense on CT compared to the surrounding brain, and because they are highly malignant, they frequently have necrotic regions and hemorrhagic alterations [7].

The standard therapeutic approach for this aggressive disease involves a multimodal approach, which includes surgery, chemotherapy (CTH), radiation (RTH), high-dose chemotherapy (HDCTH), and stem-cell rescue [8]. The management of recurrent pineoblastoma, a rare and aggressive brain tumor, is complicated, as demonstrated by the case of a 23-year-old man. The patient's clinical trajectory reveals several significant obstacles, diagnostic challenges, surgical restrictions and recurrence Issues. This case highlights the need for a multidisciplinary approach to address the complex issues associated with recurrent pineoblastoma involving radiologists, radiation oncologists, neurosurgeons, and neuro-oncologists. It also emphasizes the significance of continuing research into innovative therapeutic approaches and the requirement for customized patient care regimens.

## Case presentation

A 23-year-old male patient reported to our institution complaining of headaches and blurring vision for a month. He reported no comorbidities and no history of family cancer. On examination, he was in good condition, with no pallor or palpable lymph nodes. He had blurred vision in both eyes, while his rest cranial nerves were normal, with normal higher functions. His Power 5/5 was in all 4 limbs. He had no sensory deficits. No cerebellar signs were found; he reported having visited another hospital early that same month with similar complaints; investigations were carried out, including a brain MRI scan that revealed a well-defined heterogeneously enhancing solid cystic lesion of approximate size 2.6 × 2.4 × 2.1 cm (AP X CC X TR), noted in midline in pineal region appearing hyperintense on T2W/FLAIR and heterogeneously hypointense on T1W with hyperintense rim, showing multiple central foci of blooming on SWI with patchy areas of diffusion restriction in solid component. The patient underwent a successful endoscopic third ventriculostomy with excision of the third ventricular tumor at our hospital, which was noted as his first surgery. The histopathology results showed no evidence of malignancy; immunohistochemistry was requested to rule out low-grade glial neoplasm. He was enrolled in a regular follow-up program at our neurosurgery department.

**Fig. 1:**
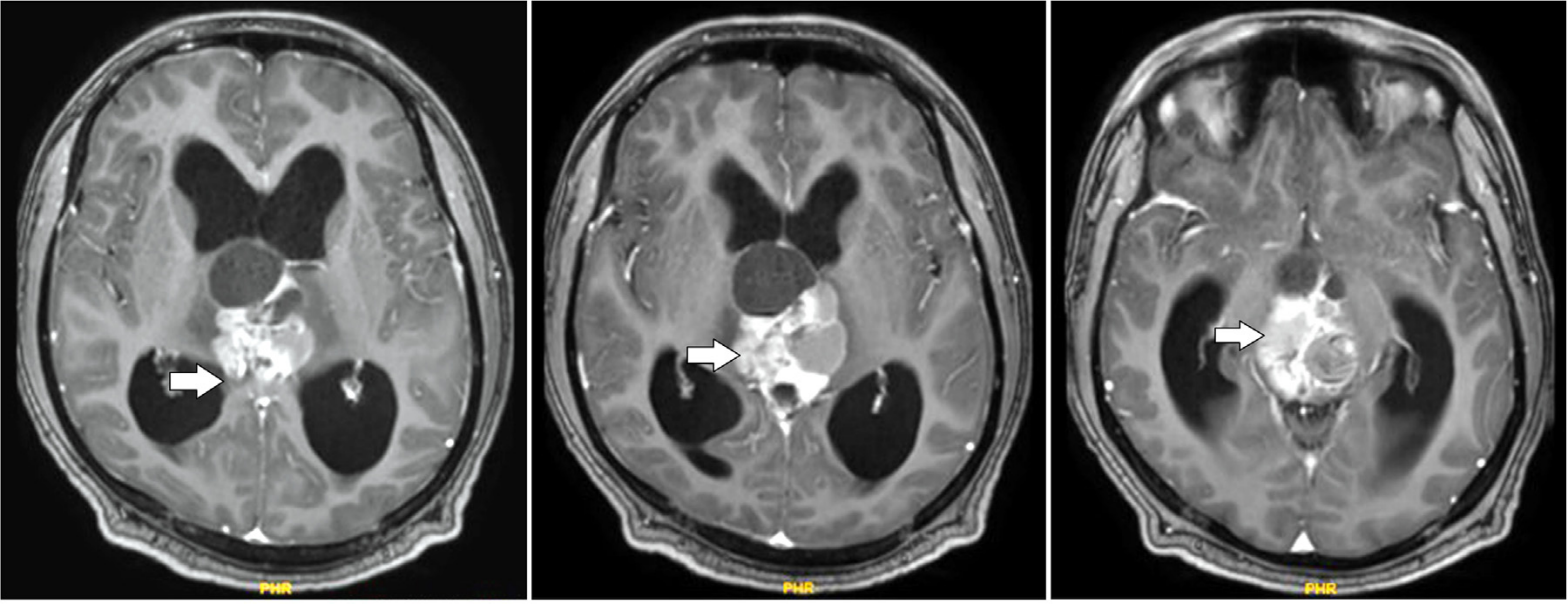
Preoperative T1 contrast MRI axial images showing the tumor in the pineal region.

Later on, the patient did a follow up brain MRI scan that reported evidence of heterogeneously enhancing solid cystic altered signal intensity lesion noted along the midline in the pineal region and third ventricle region appearing heterogeneously hyperintense on T1W1/2W1, dirty signal on FLAIR with patchy areas of diffusion restriction of solid component and showing multiple central foci of blooming on SWI. The pineal gland is not seen separately from the lesion. Most of the cystic areas showed hemorrhage within. It measures 4.9 × 3.7 × 3.5 cm. Inferiorly, the lesion compresses the midbrain. The above features suggested a residual/recurrent neoplastic lesion, likely a pineal germ cell tumor. Some features of obstructive hydrocephalus were seen along with postoperative changes in the right frontal region (Fig. 1).

The patient underwent suboccipital craniotomy with excision of pineal gland tumor (Supratentorial approach), and later, External ventricular drainage was done at our institution. Histopathology report from the sample collected reported features suggestive of pineoblastoma.

**Fig. 2:**
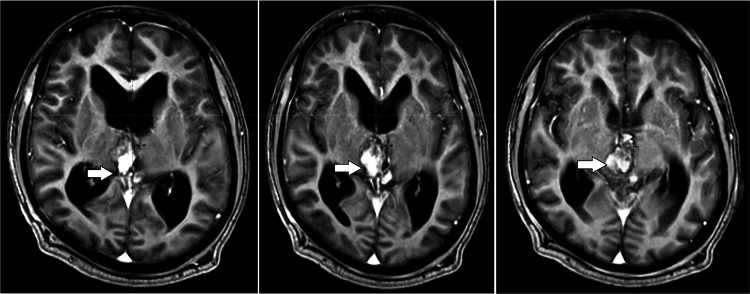
Postoperative T1 contrast MRI axial images showing the residual tumor in the pineal region after the subtotal resection of the tumor.

Afterwards, the patient returned for postoperative review and a plain brain MRI was done and revealed Postoperative status in the form of suboccipital craniotomy. CSF drainage tube noted in situ with a tip in the frontal horn of the left lateral ventricle. The postoperative extra-axial lesion in the retro cerebellar region in the midline extends to the suboccipital region in the intramuscular and subcutaneous plan. It measures 5 × 3.8 × 6.9 cm (AP into transverse into CC) in size. It appears hyperintense on T2 and hypointense on T1 and isointense on FLAIR with peripherally enhancing wall İll-defined lesion heterogeneously hyperintense lesion with peripheral hypointense rim on T2 and hyperintense at the superior aspect and hypointense at the inferior aspect on T1 seen at the postoperative site in the pineal region. The lesion shows peripheral blooming on SWI and no significant restricted diffusion. The lesion measures approximately 3.1 × 1.5 × 2 cm in size (AP × CCxT). The lesion is seen to abut and compress the superior tectal plate; Superiroly, it abuts the corpus callosum's internal cerebral vein and selenium. It is seen to compress the cerebral aqueduct. Bilateral lateral ventricles appear mildly dilated. No periventricular ooze was seen. The focal abnormal signal appears hyperintense on T2 and hyperintense on T2 with the peripheral hypointense rim, and peripheral blooming on SWI is seen in the right cerebellar region. A focal area of gliosis was seen in the right frontal lobe at the site of previously placed EVD. Mucosal thickening was seen in the left maxillary sinus. The above findings were suggestive of a recurrent pineoblastoma. Fig. 2 shows postoperative T1 contrast MRI axial mages.

### Treatment plan

**Fig. 3:**
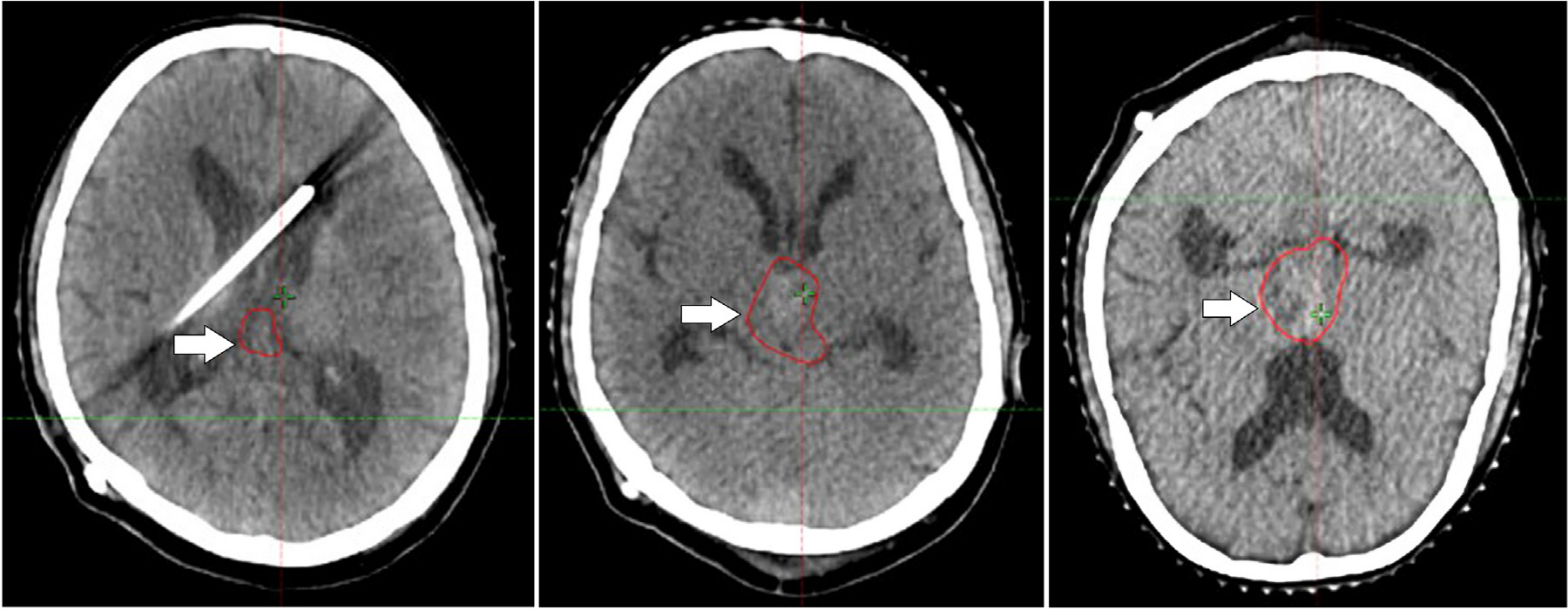
Postoperative radiotherapy planning CT Axial images showing the residual tumor in the pineal region after the subtotal resection of the tumor.

**Fig. 4:**
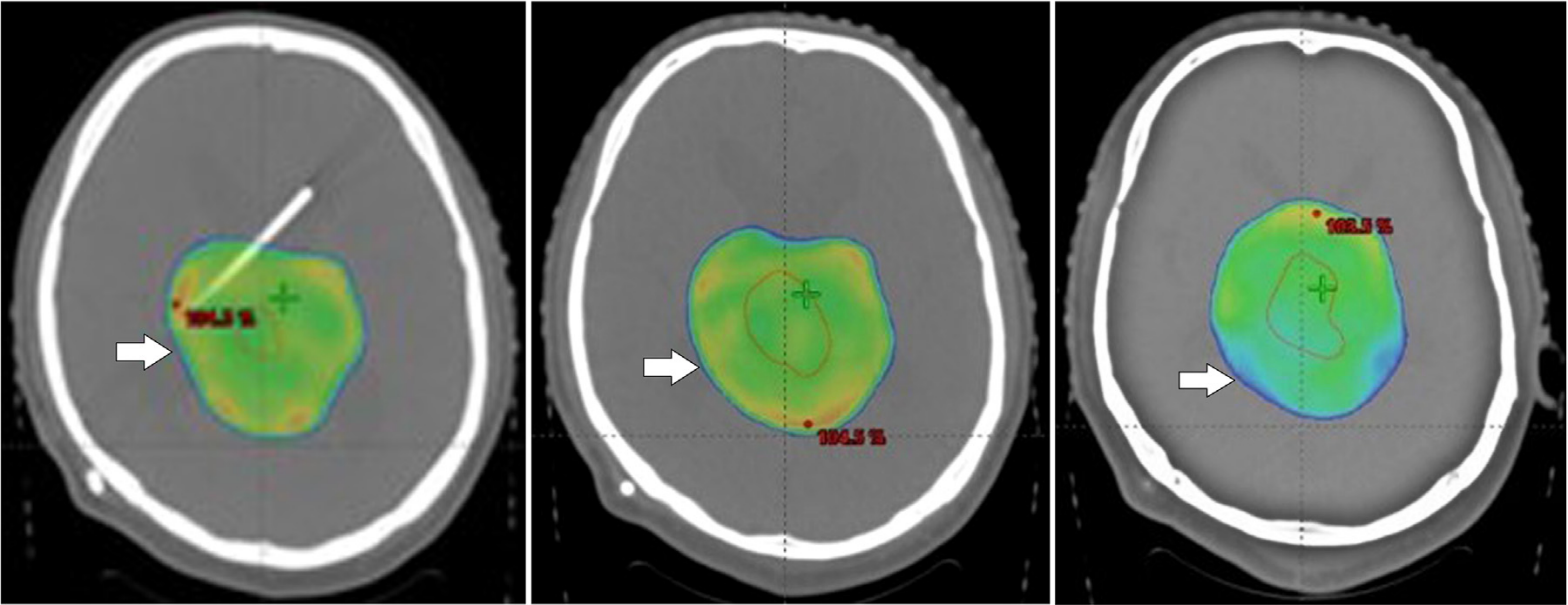
The 95% dose coverage of the planning target volume (showing inner gross tumor volume along with planning target volume margins) before delivery of the radiotherapy.

The oncologist and the multidisciplinary team devised a treatment strategy for recurrent pineoblastoma, adopting an approach similar to that used for high-risk medulloblastoma. The plan included craniospinal irradiation, administering a dose of 35 Gy in 21 fractions to the craniospinal axis followed by a boost to the gross lesion, delivering an additional 19.8 Gy in 11 fractions using VMAT (Volumetric modulated Arc therapy) to ensure precise and homogenous dose targeting of the residual lesion while sparing surrounding health tissues. Concurrent chemotherapy was initiated alongside radiotherapy to enhance therapeutic efficacy, followed by adjuvant chemotherapy to address microscopic disease and reduce the risk of further recurrence. Fig. 3, Fig. 4 illustrate radiotherapy planning CT axial images.

### Follow up

**Fig. 5:**
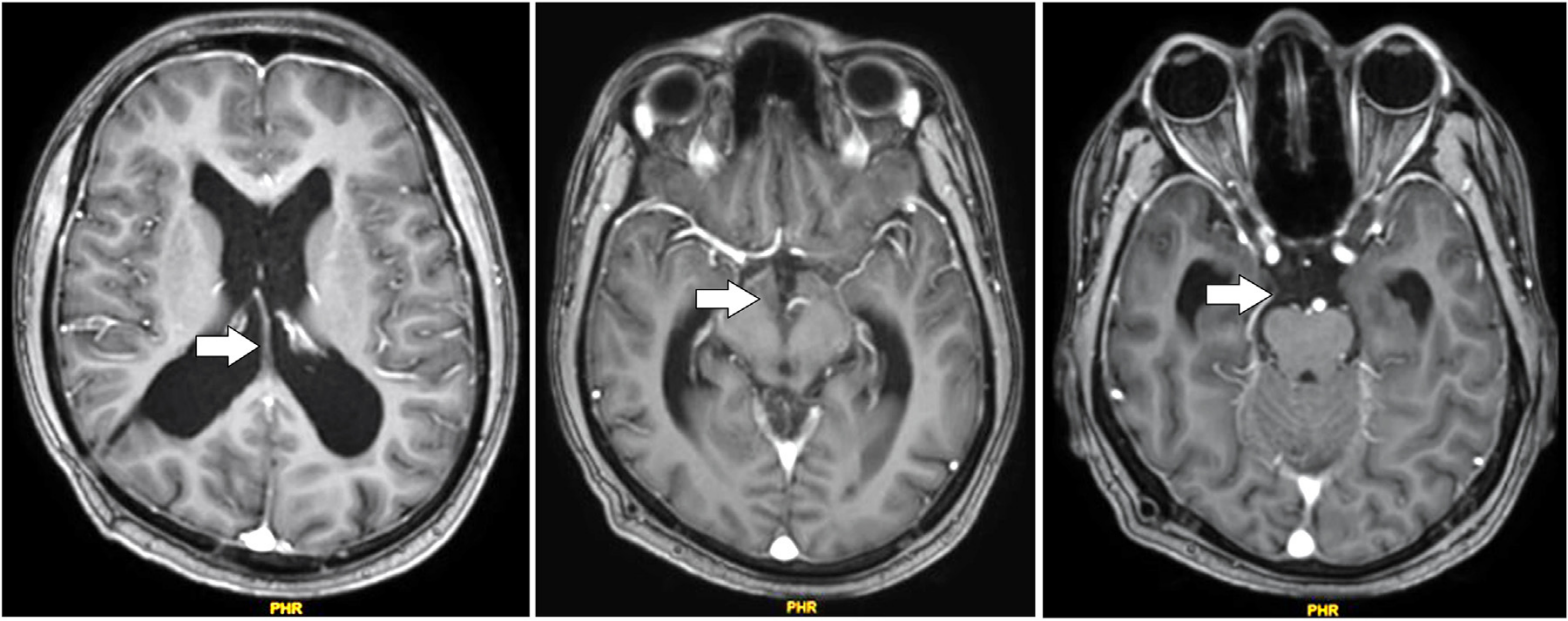
First follow up T1 contrast MRI axial images after completion of radiotherapy showing complete response with no residual tumor in the pineal region.

The patient has shown significant recovery following treatment, with no recurrence of symptoms such as headaches or visual disturbances. Postsurgical follow-ups, including neurological and ophthalmological evaluations, revealed no deficits, and imaging confirmed the absence of residual or recurrent lesions. The patient has returned to normal daily activities and continues to undergo regular follow-ups every 6 months to monitor long-term outcomes. He is advised to maintain a healthy lifestyle, adhere to prescribed medications, and seek immediate medical attention if warning signs such as persistent headaches or vision disturbances occur. Regular follow-up consultations and imaging will ensure timely detection of any recurrence and sustained well-being. This comprehensive treatment plan uses a multidisciplinary approach to minimize treatment-related toxicity while optimizing local and systemic management. Fig. 5 shows the first follow-up T1 contrast MRI axial images.

## Discussion

Pineoblastomas are sporadic and highly malignant embryonal tumors graded by the World Health Organization as Grade IV. Although they are mainly identified in children, they can be fatal to younger adults as well [9]. Pineoblastomas are less common in this age range; the patient in this instance, a 23-year-old man, represents an uncommon demography. Obstructive hydrocephalus secondary to the mass in the region of the pineal gland is seen with common presentations of the patient complaining of headache, nausea, and visual disturbances. According to Gener et al. [10], it has been observed that 70%-80% of patients have signs of increased intracranial pressure. Considering the above, our patient presented with progressively worsening headaches and blurry vision.

Surgery reduces the symptoms associated with the increased intracranial pressure and provides tissue samples for histopathological examination apart from reducing tumor bulk. However, the intervention is very morbid and mortal with complications that might be detrimental to the quality of life, as depicted in a case report by Gaito et al. [11]. Our patient received surgery twice, and histopathological results were noted.

A study by David N. Louis et al. observed that while pineoblastomas comprise sheets of poorly differentiated embryonal neoplastic cells, their histology is not unique. Therefore, the first crucial step in ruling out other nonlinear embryonal cancers, particularly medulloblastomas, is confirming that the tumor is in the spinal area [12]. A histopathological examination of our case revealed a fragment of a neuroepithelial tumor of variable cellularity with perivascular pseudorosettes and Homer-Wright rosettes. Some fragments were hypocellular with fibrous stroma and tumor cells forming pineocytomatous rosettes. Immunohistochemistry (IHC) revealed that the tumor cells were strongly and diffusely positive for synaptophysin favoring PB.

Neuroimaging is integral to diagnostic evaluation, surgical strategizing, and postoperative monitoring of patients presenting with pineal masses. Even though neuroimaging frequently yields conflicting results on the type of lesion, both CT and MRI are helpful concerning the pineal tumor's location, dimensions, and morphology [7]. Hypo-intensity to iso-intensity on T1 weighted images and iso-intensity to hyper-intensity on T2 weighted images are the typical MRI characteristics of PB, as also seen in our case.

Pineoblastoma often appears hyperdense on CT scans with minimal or absent calcifications, unlike pineocytomas. It frequently causes obstructive hydrocephalus by compressing the cerebral aqueduct. MRI shows hypointense to isointense on T1, hyperintense on T2, and substantial, heterogeneous postcontrast enhancement. Diffusion-weighted imaging (DWI) reveals restricted diffusion, and MR spectroscopy demonstrates a high choline peak with a low NAA peak, indicating high cell turnover. The tumor often exhibits leptomeningeal spread, necessitating spinal MRI evaluation. An appropriately defined intervention in PB has not yet been established due to a very low incidence rate, especially in adults, and very few reported cases with no follow-up and outcomes [13].

Maximum surgical resection is recommended for adults with PB. It should be followed by adjuvant craniospinal radiotherapy, with a boost to the whole posterior fossa, and may be in conjunction with chemotherapy challenges in the resection of these lesions. Resecting pineoblastomas is challenging due to their deep location**,** requiring complex surgical approaches while avoiding injury to critical structures like the brainstem, deep veins, and tectal plate. Their high vascularity increases the risk of significant bleeding**,** and strong adhesions to surrounding tissues make complete resection difficult**.** Many patients present with obstructive hydrocephalus, often necessitating CSF diversion before or during surgery. Additionally, the tumor's tendency for leptomeningeal spread limits surgical benefits, making multimodal treatment with chemotherapy and radiotherapy essential**.** Postoperative risks include Parinaud's syndrome, hydrocephalus recurrence, and neurological deficits.

Tumors of the pineal region vary in recurrence risks and patterns of spread [14]. Tumors of the pineal region have followed various patterns of recurrence and metastasis. Some literature reports mention that radiotherapy managed to control the tumor with improved survival [15]. The recurrence within months of the first surgery shows that the disease is aggressive. Our patient was initially taken up for subtotal resection with adjuvant therapy, resulting in remission. Despite aggressive treatment, the prognosis remains poor. According to Chu et al., the 5-year survival rate is about 58% in young adults, and recurrence risk is high in those with subtotal resections or leptomeningeal spread [16]. Additionally, a study by Kang et al. [17] found that patients who get chemotherapy and radiation therapy can have long-term survival.

This case demands a multidisciplinary management approach in complex recurrences. An early diagnosis and intervention into recurrence could improve the outcomes; hence, there is an emergent need for novel therapies and molecular diagnostics to address the characteristic therapeutic resistance of recurrent pineoblastomas.

## Conclusion

This case of recurrent pineoblastoma in a 23-year-old male stresses the aggressiveness of the disease, the importance of molecular profiling for prompt diagnosis and the problems in managing recurrences. With initial remission, the recurrence indicates long-term surveillance and individualized management. Standard treatments are only marginally successful, necessitating new therapies. Hence, further research is necessary to optimize diagnostic and therapeutic strategies, especially in young adult patients.

## Ethical approval

A consent form has been taken before taking the patient data.

## Patient consent

The patient concern form has been taken before starting of the study.

## CRediT authorship contribution statement

**Lydia Kiwanuka:** Writing – review & editing, Conceptualization, Data curation, Visualization. **Induni Nayodhara Weerarathna:** Writing – review & editing, Conceptualization, Data curation, Visualization. **Neha Rahul:** Supervision. **Manishimwe Jules:** Writing – review & editing. **Anurag Luharia:** Supervision. **Zahir Quazi:** Supervision.
